# Parental Barriers and Sociodemographic Disparities in Childhood Vaccination Post-COVID-19 in Tennessee

**DOI:** 10.3390/vaccines13050452

**Published:** 2025-04-24

**Authors:** Sanjaya Regmi, Elizabeth Sowell, Chenoa D. Allen, Benjamin E. Jones, Nan M. Gaylord, Victoria Niederhauser

**Affiliations:** 1 College of Nursing, The University of Tennessee, Knoxville, TN 37996, USA; 2 Social Work Office of Research and Public Service, The University of Tennessee, Knoxville, TN 37996, USA

**Keywords:** vaccination barriers, childhood immunization, vaccine hesitancy, vaccine concerns, COVID-19, vaccine disparities, routine immunization, Tennessee

## Abstract

Introduction: The COVID-19 pandemic disrupted routine childhood vaccinations schedules, posing significant challenges among underserved communities. Understanding how different sociodemographic groups in Tennessee perceive and navigate childhood vaccination barriers is critical for developing strategies to improve vaccination rates and reduce vulnerability to vaccine-preventable diseases. Methods: A cross-sectional survey was conducted to explore barriers to vaccination across diverse sociodemographic groups in Tennessee. Data were collected from caregivers/parents of children aged 18 years and younger across all 95 counties in Tennessee at community events and through partnerships with schools and other local organizations. Parental responses were analyzed to identify barriers in access, concern, and importance domains. The distribution of barriers across different sociodemographic groups such as race, income, education level, and insurance status was identified. Descriptive statistics, non-parametric tests, and log-binomial regressions were used to address the research objectives. Results: This study found that the most prominent barriers to childhood vaccination were concerns regarding vaccine safety and side effects. Significant differences in vaccine barriers were observed across racial and ethnic groups for access barriers (*p* < 0.001), concern barriers (*p* = 0.006), and importance barriers (*p* < 0.001). Parents with lower education levels, children without health insurance, and lower-income families faced disproportionate challenges across two of the three barrier domains studied (access and perceived importance of vaccines). Additionally, concern barriers (aPR = 0.998, *p* < 0.001) and importance barriers (aPR = 0.997, *p* < 0.001) were strongly associated with the parent-reported prevalence of up-to-date vaccination status. Conclusions: Addressing parental vaccination barriers related to concern, access, and perceived importance is crucial, particularly for underserved populations including low-income families, uninsured parents, racial/ethnic minorities, and those with limited education. A sustained, equity-focused approach integrating scientific communication, community engagement, and policy interventions is essential for increasing vaccine uptake and ensuring equitable vaccination access.

## 1. Introduction

Childhood vaccination is a cornerstone of public health, significantly reducing the burden of infectious diseases and improving survival rates [1]. However, many parents harbor persistent concerns and encounter systemic barriers that delay or prevent the timely vaccination of their children. Furthermore, the COVID-19 pandemic has changed vaccination concerns and the perceived importance of routine childhood vaccinations among parents [2,3]. The vaccination rates for key vaccines, such as measles, mumps, and rubella (MMR), diphtheria, tetanus, pertussis (DTaP), and polio (IPV), have notably declined in recent years. During the 2022–2023 school year, vaccination coverage among kindergarten-aged children in the U.S. dropped to approximately 93%, from 95% in the 2019–2020 school year [4]. Tennessee Immunization Information System (TennIIS) county data show a 2–26% drop in early childhood vaccinations during the COVID-19 pandemic [5]. This decline has been particularly concerning in Tennessee, which has consistently ranked among the bottom ten states in health outcomes since the 1990s [6]. Given the longstanding health disparities in Tennessee, vulnerable communities remain at a heightened risk of vaccine-preventable diseases (VPDs), such as measles [7,8].

Childhood vaccine barriers reflect the challenges faced by parents in accessing and receiving vaccinations for their children. These hardships influence vaccine intentions and, in turn, affect vaccine behavior [9]. The theory of planned behavior posits that behavioral intentions are influenced by attitudes, subjective norms, and perceived behavioral control [10]. Guided by this framework, we categorized barriers to childhood vaccination into three primary domains: concern, importance, and access. Concern reflects parental attitudes toward vaccination, including beliefs about vaccine safety and efficacy. Importance aligns with subjective norms, capturing perceived social pressures and cultural expectations regarding vaccination. Access pertains to perceived behavioral control, addressing logistical and systemic factors that facilitate or impede vaccination [11]. Together, these domains provide a comprehensive framework for examining the multifaceted barriers that shape parental vaccination decisions. Moreover, parental vaccine decisions are deeply embedded within broader social determinants of health, which shape attitudes, access, and perceived importance through lived experiences of inequality, mistrust, and marginalization [12,13,14]. Access barriers include transportation difficulties, clinic unavailability, long wait times, lack of information, and economic hardships, disproportionately affecting low-income families and rural communities, where healthcare resources are often limited [15]. Concern barriers arise from fears regarding vaccine safety, side effects, and ingredients, which have been significantly heightened by the pandemic. The proliferation of misinformation, particularly through social media and informal networks, has amplified these fears and contributed to vaccine-related uncertainty and distrust [16]. This distrust in vaccines is particularly entrenched in communities with histories of exclusion and medical mistreatment, where historical injustices and systemic inequities have fueled skepticism toward public health campaigns [14,17]. Misinformation of vaccine ingredients and vaccine safety has worsened the fears of vaccines [18], with anxiety about the COVID-19 vaccine spilling over into routine childhood vaccinations [19,20,21]. This erosion of trust, fueled by misinformation, has weakened the impact of public health campaigns and contributed to childhood vaccine hesitancy in certain communities. Importance barriers occur when parents question the necessity of vaccination, particularly if they perceive their children to be at low risk of contracting VPDs [22]. Cultural norms, social networks, political skepticism [18], and mistrust of medical institutions strongly influence beliefs regarding the importance of vaccination in children [23].

Parents facing barriers—especially those shaped by structural determinants such as poverty, rural isolation, and lack of healthcare access—are less likely to have their children vaccinated on time [24]. Parents who have heightened safety concerns, often influenced by misinformation, are more likely to delay or avoid vaccinations altogether [9]. These parental barriers to vaccination vary across sociodemographic groups [25]. Access barriers are significantly influenced by family income, clinic availability, transportation, health insurance, and rural/urban residency [26]. Concern barriers and the perceived importance of childhood vaccination are shaped by parental education level, race, and health insurance, with higher education levels and insurance coverage generally associated with lower vaccine hesitancy [27].

Given the complex interplay between vaccine barriers and inequities, addressing parental vaccine barriers requires a nuanced understanding of the sociodemographic characteristics that influence these challenges. This study examined parental barriers to childhood vaccination across three domains, based on the theory of planned behavior, and analyzed the social determinants underlying these barriers. This study addresses the following key questions: (1) What are the parental barriers to vaccination in Tennessee? (2) What is the distribution of parental vaccine barriers across different sociodemographic groups? (3) Are these vaccine barriers linked to the up-to-date vaccination status of children? By analyzing the distribution of vaccine barriers across different sociodemographic groups and assessing their impact on parent-reported vaccination status, this study aimed to provide a fresh understanding of the landscape of childhood vaccination barriers and their distribution across various sociodemographic groups in the post-pandemic era. These efforts are essential for developing targeted, community-specific childhood vaccine interventions.

## 2. Materials and Methods

### 2.1. Survey Methods

This study surveyed the parents and caregivers of children aged 18 years or younger using a convenience sampling approach. Data were collected between July 2022 and May 2024, encompassing all 95 counties in Tennessee. The highest proportion of participants were from Lawrence county (12.8%), followed by Carter county (9.7%), with each of the remaining counties contributing less than 5% of the sample. To address potential bias from this uneven geographic distribution, we conducted a sensitivity analysis (see statistical analysis). Sixteen community-registered nurse navigators (CRNNs), actively working in minimizing vaccine barriers, facilitated the data collection. Surveys were distributed at diverse community outreach events, including health fairs, head start programs, and community baby showers. At these events, CRNNs explained the project and invited eligible parents to voluntarily participate by completing a self-administered paper survey on site. Additionally, surveys were disseminated through collaborations with educational institutions such as schools, daycare, and preschools, where paper surveys were sent home for parents to complete and return. Surveys were also collected online. QR code access to the online survey was posted at partner institutions, such as local health departments. Access to the online survey was restricted to eligible participants and could only be reached via the QR code. The survey was available in English (99.1% of responses) and Spanish (0.9% of responses; 3.9% of Tennessee’s population speak Spanish). Of the completed surveys, 13.7% were submitted online via Qualtrics, whereas 86.3% were completed using paper forms. The estimated completion time for each survey was 5–10 min. While the participants did not receive financial compensation, the CRNNs provided vaccine-related education to those who expressed interest.

### 2.2. Measures

#### 2.2.1. Parental Vaccine Barriers

This study employed the validated Search for Hardships and Obstacles to Shots (SHOTS) questionnaire to assess parental vaccine barriers across three core domains: access, concern, and importance [11]. The SHOTS instrument was developed based on the theory of reasoned action, which emphasizes attitudes, social influences, perceived consequences, habits, and facilitating conditions in shaping behavior. While grounded in the theory of reasoned action, the SHOTS instrument also aligns conceptually with the theory of planned behavior (TPB), reflecting core constructs such as attitudes (concern), subjective norms (importance), and perceived behavioral control (access). The tool captures behavioral, cognitive, and emotional dimensions of vaccine decision-making and has been translated into multiple languages, demonstrating high reliability and cross-cultural applicability in diverse settings [28,29,30]. In addition to the SHOTS survey questionnaire, additional sociodemographic questions and childhood vaccination status questions were asked in the survey. The SHOTS survey consists of 23 questions: 12 for access-related barriers, 6 for concern-related barriers, and 5 for importance-related barriers (Table 1). Responses were recorded on a scale ranging from 0 (not a problem) to 4 (very big problem). The total score for each construct was derived by summing the participants’ responses across all corresponding items. The possible score ranges were access (0–48), concern (0–24), and importance (0–20). The internal consistency of the survey questions was excellent, with Cronbach’s alpha for all survey questions being 0.944 for access questions, 0.941 for concern questions, and 0.943 for importance questions 0.920 (Table 1). To assess the structural validity of the instrument, a confirmatory factor analysis (CFA) was conducted, and the findings are presented in the Results Section.

#### 2.2.2. Parent-Reported Vaccine Status

Parents/caregivers were asked, ‘Is the immunization status of your youngest child up-to-date?’ with response options of no, yes, or unsure. In this study, up-to-date vaccination status refers to children having received all vaccines recommended for their age group according to the CDC’s Advisory Committee on Immunization Practices (ACIP) guidelines [31]. However, the parents were not explicitly provided with a comprehensive list of vaccines for the survey question. For reporting purposes, not up-to-date vaccination status is classified as under-vaccination.

#### 2.2.3. Sociodemographic Variables

Age of Youngest Child: Participants were asked to report the age of the youngest child residing in their household. Based on the CDC guidelines, responses were categorized into three developmental stages: early childhood (0–2 years), preschool age (3–6 years), and school age (7–19 years). Parental Education Level: Participants provided information on their highest level of education. Responses were grouped into four categories: less than high school, high school diploma, some college, and college degree. Race/ethnicity: Participants selected one or more of the racial/ethnic categories from the following options: non-Hispanic white, non-Hispanic African American, Hispanic, Middle Eastern, North African, Native American, American Indian, Alaska Native, Indigenous, Asian, Native Hawaiian, Other Pacific Islander, or any other race/ethnicity. The “other/multiracial” category included participants who selected more than one race or ethnicity or provided an open-text response not aligned with the standard categories. Household Income: Participants were asked to report their total household income for 2021, inclusive of all family members. Income levels were classified based on federal poverty guidelines for a two-person household: less than USD 20,000 (0–99% FPL), USD 21,000–40,000 (100–199% FPL), USD 41,000–75,000 (200–400% FPL), and above USD 76,000 (>400% FPL). Health Insurance Coverage: Participants were asked whether their child had any form of health insurance, including private or government-funded programs such as Medicare, TennCare, or Indian Health Service. The responses were yes, no, or unsure. Geographic Location: Participants’ residential counties were classified based on the U.S. Department of Agriculture Economic Research Service into two categories: non-metro and metro.

### 2.3. Statistical Analyses

A total of 9922 survey responses were collected. After data cleaning, responses with more than 50% missing values were excluded, yielding a sample of 9343 participants. Within this sample, 389 parents reported uncertainty regarding their children’s vaccination status. A sensitivity analysis was conducted to assess model performance under two different scenarios: (1) responses marked as “unsure” were recategorized into the “no/unsure” category, and (2) “unsure” responses were excluded from the analysis. Comparative model assessments indicated that excluding the “unsure” responses improved model fit, yielding a lower deviance (739.473 vs. 921.730) compared to the model that recategorized “unsure” responses. Given the superior fit of the exclusion model, the final analysis was conducted with a final sample of 8954 participants (90.24% of the survey). To examine potential bias due to geographic overrepresentation, we conducted a sensitivity analysis using the Mann–Whitney U-test to compare key variables between participants from the two most represented counties (Lawrence and Carter) and those from all other counties. We found no significant differences in the key variables (*p* > 0.05). To assess the dimensional structure of the SHOTS instrument, we conducted a confirmatory factor analysis using Mplus 8.10 (Muthén & Muthén, Los Angeles, CA, USA). The model tested a three-factor solution representing access, concern, and importance. Fit was evaluated using standard indices (CFI, TLI, RMSEA, SRMR), and factor loadings were inspected to assess item–factor relationships.

Several statistical analyses were conducted to examine the data comprehensively. Frequencies and percentages were used to summarize sociodemographic distributions and under-vaccination rates across groups. Descriptive statistics, including the mean and standard deviation, were calculated for each item within the three barrier categories: access, concern, and importance. The response scale ranged from 0 to 4, with higher values indicating higher barriers. Internal consistency for each category was assessed using Cronbach’s alpha to evaluate reliability. The association between sociodemographic variables and parental vaccination barriers was assessed using the Kruskal–Wallis test. For each sociodemographic variable, the median, interquartile range (IQR), and mean values were reported to illustrate the relative differences in barrier levels. A post hoc multiple comparison test was performed to identify homogeneous subsets of sociodemographic groups. Finally, a log-binomial regression model was used to examine the relationship between the up-to-date vaccination status and parental vaccine barriers (access, concern, and importance). Before fitting multivariable log-binomial regression models, we assessed multicollinearity among the predictors using variance inflation factor (VIF) diagnostics. All VIF values ranged from 1.006 to 1.276, well below the conventional threshold of 5, indicating no significant collinearity among the variables. As a result, all covariates were retained in the final adjusted models. The adjusted prevalence ratios (aPRs) and 95% confidence intervals (CIs) were calculated. The model was adjusted for race, family income, health insurance status, and parental education level to control for potential confounders. Statistical significance was set at *p* < 0.05. All analyses were performed using the IBM SPSS version 28 (IBM Corp., New York, NY, USA).

## 3. Results

### 3.1. Participants

Among the total participants (*N* = 8954), the largest proportion were parents or caregivers of school-aged children (7–18 years; 42%). The majority of parents had at least a high school education (92.6% vs. 89.6% statewide) and identified as non-Hispanic white (82.4% vs. 78.4% statewide). The household income distribution showed that 44.1% of participants fell below USD 40,000 income. Most participants reported having health insurance (93.8% vs. 94.6% statewide), and the sample was nearly evenly split between non-metro (51.9%) and metro counties (48.1%) (Table 2).

### 3.2. Parental Barriers to Childhood Vaccination in Tennessee

Among the 23 itemized barriers of the SHOTS survey, all 6 barriers in the concern domain had the highest mean parental barrier scores. For example, the statement “If something bad happened to my child after a shot, I would feel like it is my fault” had the highest mean score (mean = 1.30, SD = 1.57). Additionally, safety concerns, such as “I worry about what’s in the shot”, had the second-highest mean score (mean = 0.96, SD = 1.36), and “I worry my child might get sick from the shot” had the third-highest mean score (mean = 0.91, SD = 1.29). Conversely, access barriers such as “I didn’t know where to take my child to get his/her shots” (mean = 0.20, SD = 0.73) and “Getting my child in for shots is too much trouble” (mean = 0.22, SD = 0.74) had the lowest mean scores. Among importance barriers, health reasons of children had the lowest reported mean score: “My health care provider told me not to get my child his/her shots” (mean = 0.21, SD = 0.77) (Table 1).

### 3.3. Confirmatory Factor Analysis

**Figure 1:**
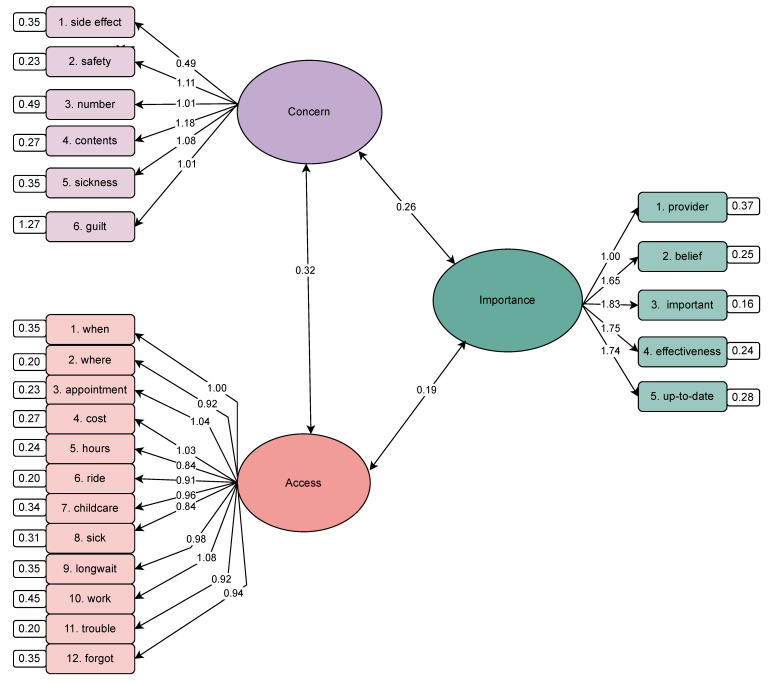
Confirmatory factor analysis of the SHOTS instrument demonstrating the three-factor structure: access, concern, and importance.

A confirmatory factor analysis was conducted to validate the three-factor structure of the SHOTS instrument, reflecting access, concern, and importance barriers. The model demonstrated acceptable to good fit (χ^2^(227) = 10,948.13, *p* < 0.001; CFI = 0.935; TLI = 0.928; RMSEA = 0.074 (90% CI: 0.072–0.075); SRMR = 0.052). All standardized factor loadings were statistically significant (*p* < 0.001), ranging from 0.49 to 1.83. The access factor included 12 items with loadings ranging from 0.84 to 1.08. The concern factor included six items, with loadings from 0.49 to 1.11. The importance factor included five items, with loadings from 1.00 to 1.83. Inter-factor correlations were moderate: access–concern (r = 0.32), access–importance (r = 0.19), and concern–importance (r = 0.27), supporting construct distinctiveness (Figure 1).

### 3.4. Disparities of Vaccine Barriers Among Different Sociodemographic Groups in Tennessee

Table 3 shows the childhood vaccine barriers across various sociodemographic groups. The age of the youngest child was significantly associated with access barriers (*p* < 0.001). Although statistically significant, the differences in mean access scores between age groups were small, suggesting limited practical impact on vaccine access. Parents of early-age children (0–2 years) reported the highest access barrier score (0 [0–3], mean = 3.4). The concern and importance barriers did not significantly differ across age groups. Parental education was significantly associated with access barriers (*p* < 0.001) and importance barriers (*p* < 0.001). Parents with less than high school education reported the highest access barrier score (0 [0–4], mean = 4.1) and importance barrier score (0 [0–1], mean = 1.9). In contrast, college graduate parents had the lowest access barrier (0 [0–2], mean = 2.3) and importance barrier scores (0 [0–0], mean = 1.3). These differences of 1.8 points on the access scale (range 0–48) and 0.6 on the importance scale (range 0–18) may represent modest but meaningful disparities, particularly in the context of cumulative barriers in underserved populations. The concern barriers did not vary significantly across education levels. Significant differences were observed across racial and ethnic groups for all barriers: access barriers (*p* < 0.001), concern barriers (*p* = 0.006), and importance barriers (*p* < 0.001). The other/multiracial group exhibited the highest barriers across all categories, with an access barrier score of (4 [0–12], mean = 7.7), concern barrier score of (8 [0–20], mean = 10.0), and importance barrier score of (0 [0–12], mean = 5.8). Their consistently higher barrier scores across all domains may reflect intersecting structural barriers, cultural exclusion, or a lack of targeted communication in public health efforts. In contrast, non-Hispanic white parents had the lowest access barrier score (0 [0–2], mean = 3.0). Income level was significantly associated with access barriers (*p* < 0.001) and importance barriers (*p* < 0.001). Families in the lowest income category reported the highest access barrier score (0 [0–4], mean = 4.5). Conversely, families earning the highest income category had the lowest access barrier score (0 [0–1], mean = 2.1). Parents without health insurance reported significantly higher access barriers (*p* < 0.001) and importance barriers (*p* < 0.001) than insured parents. Concern barriers were not significantly different across the health insurance status of children. Parents living in metro areas reported slightly higher importance barriers (*p* = 0.046) and access barriers (*p* < 0.001) than in non-metro areas (Table 3). While statistically significant, these geographical differences may have limited public health relevance unless supported by other structural disparities (e.g., transportation and health system complexity).

### 3.5. Association Between Categories of Parental Vaccine Barriers and Parent-Reported Up-to-Date Vaccination Status

Table 4 explores the associations between parental vaccine barriers and parent-reported up-to-date vaccination status. The concern barrier was significantly associated with a lower prevalence of up-to-date vaccination status (B = −0.002, aPR = 0.998, 95% CI [0.997, 0.998], *p* < 0.001). This indicates that for each unit increase in the concern barrier score, the prevalence of a child being up to date on vaccinations decreased by 0.2%. Similarly, the importance barrier also showed a significant negative association with up-to-date vaccination status (B = −0.003, aPR = 0.997, 95% CI [0.996, 0.998], *p* < 0.001), suggesting that each unit increase in the importance barrier score was associated with a 0.3% decrease in the prevalence of a child being up to date on vaccinations. In contrast, access barriers did not have a meaningful impact on the up-to-date vaccination status of children (B = 0.001, aPR = 1.001, 95% CI [1.000, 1.002]) (Table 4).

## 4. Discussion

This study examined parental vaccine barriers in Tennessee using the SHOTS instrument. The barriers are grouped into three categories based on the theory of planned behavior: concern (attitudes), importance (subjective norms), and access (perceived behavioral control). The current study adds to the psychometric validation of the SHOTS instrument by confirming its three-factor structure using CFA. Factor loadings were strong, and model fit was acceptable, indicating that the access, concern, and importance domains capture distinguishable constructs of vaccine barriers. Our findings highlight significant disparities in vaccine barriers across different sociodemographic groups, including race/ethnicity, household income, education level, health insurance status, and metro versus non-metro residences. These results offer insights into areas in which targeted interventions can enhance vaccine confidence and accessibility.

### 4.1. Parental Barriers to Childhood Vaccination

This study identified concern of side effects and vaccine safety as the most prominent parental barriers to routine childhood vaccinations. Statements reflecting fears of personal culpability for adverse effects, concerns about vaccine ingredients, and concern about vaccine-related illnesses had the highest barrier scores. These findings suggest that, while logistical barriers exist, concerns about vaccine safety and side effects dominate parental hesitation. These findings align with prior research emphasizing the significant role that vaccine safety concerns play in parental decision-making [32,33]. Heightened parental concerns are likely due to misinformation during the COVID-19 pandemic, which has eroded public trust in healthcare and governmental institutions [33,34]. Furthermore, incorporating the COVID-19 vaccine into routine schedules may have intensified concerns regarding routine vaccinations [35,36].

This study contributes to the growing literature by highlighting that safety-related issues, such as concerns about vaccine ingredients (“worrying about what’s in the shot”) and side effects (e.g., “feeling at fault if something bad happened after a shot”), are paramount among parental barriers. Increased anxiety about making the wrong health decisions, especially in an era of accessible conflicting information, can exacerbate these issues. Studies have shown that minority groups are disproportionately targeted and susceptible to misinformation and political skepticism, which are significant factors contributing to vaccine hesitancy [19,37]. Future research should explore safety and responsibility concerns more deeply, investigating their relationship with vaccine hesitancy and vaccine uptake. Moreover, leveraging parents’ genuine concerns about their children’s health could be an opportunity to disseminate accurate information based on the scientific and global outcomes of routine vaccinations.

An effective strategy to address concern barriers could involve identifying and directly addressing parental concerns. Public health professionals and vaccine providers might use evidence-based screening tools like the SHOTS survey [11] to specifically target parental concerns. Additionally, initiatives such as brief evidence-backed educational interventions and community health worker programs could be successful in engaging vaccine-hesitant families [38]. Behavioral techniques such as motivational interviewing [39], face-to-face interventions for educating parents [40], and culturally relevant storytelling [21] may help reframe risk perceptions and build trust in routine vaccination. Furthermore, consistent and transparent communication from trusted sources such as primary care physicians [21], community-registered nurse navigators [41], school nurses [42], and faith leaders [43] should be leveraged to address misinformation and promote vaccine literacy. These efforts are vital for building trust and facilitating dialog in communities affected by misinformation and political skepticism, thus improving healthcare networks and enhancing vaccination coverage among minority groups.

### 4.2. Disparities in Childhood Vaccine Barriers Across Sociodemographic Groups

This study elucidated substantial sociodemographic disparities in all domains of parental vaccine barriers across Tennessee, notably among racial minorities, lower-income families, parents with lower educational attainment, and children without health insurance. This underscores the persistent challenges in vaccine access and equity, aligned with broader U.S. studies [36,44]. Factors such as historical mistrust in medical institutions, systemic inequities, and socioeconomic challenges profoundly contribute to vaccine hesitancy among these groups [45,46]. The findings from this study uncovered an unexpected trend: despite assumptions that non-metro areas face greater healthcare challenges, metro families reported higher vaccine barriers. The strain on metro healthcare systems as a post-pandemic effect may have exacerbated access issues in these settings [47]. However, this pattern should be interpreted with caution as it may reflect local context, sample variability, or other unmeasured factors. The comprehensive approach of this study provides a nuanced understanding of childhood vaccine barriers across different sociodemographic groups, facilitating the design of targeted public health interventions. Effective strategies to remove disparities may include mobile clinics and outreach camps for vaccination [25] in the identified pockets of these sociodemographic groups. Additionally, community-based interventions should incorporate multilingual and culturally appropriate educational and communication strategies tailored to the targeted groups [38]. Partnerships with local organizations, faith-based institutions, and trusted messengers may further enhance outreach and reduce perceived barriers in historically underserved populations [43].

### 4.3. Association Between Childhood Vaccine Barriers and Vaccination Status

This study highlights that concerns about vaccine safety and side effects, along with the less perceived importance of vaccines, are significant predictors of up-to-date vaccination status. These findings underscore the influence of knowledge gaps, misinformation, and structural healthcare inequalities on vaccine uptake [19,20,21,37]. Consistent with the literature, concerns related to vaccine safety and side effects were particularly influential in driving vaccine hesitancy [20,21]. Additionally, politically motivated skepticism about vaccine importance remains one of the most significant and persistent barriers to vaccine uptake [25]. Furthermore, access barriers, including challenges with scheduling and limited clinic availability, affect vaccine uptake, emphasizing the need for improved healthcare logistics [15,48,49]. The results of this study suggest the need to increase vaccine confidence within the community, especially among underserved populations. Integrated interventions that address access barriers (such as mobile vaccination units), concern-related barriers (such as tailored educational efforts), and perceived importance (such as personal risk communication) are critical for improving vaccine uptake. One effective strategy could be the use of psychological inoculation campaigns to build resilience against vaccine misinformation, both online and in person [50]. Additionally, public health efforts should continue to address vaccine hesitancy along with access issues by increasing the number of Vaccines for Children (VFC) clinics and offering more flexible clinic hours [51] combined with community outreach programs.

### 4.4. Limitations and Areas for Future Work

This study has several limitations that should be considered when interpreting the findings. First, reliance on parent/caregiver-reported vaccination status introduces the potential for recall bias. This limitation may be more pronounced among parents with lower health literacy or education levels, potentially resulting in differential misclassification. Second, participant recruitment was not randomized, as individuals were largely drawn from public events (e.g., head start programs) and institutional settings (e.g., schools). This may have introduced selection bias, with individuals more interested in vaccination potentially more likely to participate. Third, the sample included a slightly higher proportion of participants who identified as non-Hispanic white (82.4%) compared to the statewide estimate (78.4%) [52], which may limit the applicability of the findings to more racially and ethnically diverse populations within Tennessee. Despite these limitations, a reasonable representation of Tennessee residents in the survey offers valuable insights into the vaccination barriers in the state. Given the use of convenience sampling, the generalizability of the findings is limited. However, many of the observed patterns—such as concerns about vaccine safety, the impact of misinformation, and disparities across sociodemographic groups—are consistent with national trends. Additionally, this study’s focus on Tennessee-specific cultural and regional factors further limits the generalizability of the findings to other areas. Nonetheless, the findings may be cautiously applicable to other regions in the United States with similar rural–urban dynamics, health system characteristics, and underserved populations. Despite strong psychometric properties, the SHOTS instrument may not fully capture region-specific vaccine hesitancy dynamics. Cultural, historical, and political contexts—such as structural mistrust or politicized vaccine discourse—may require further adaptation or item refinement in future research to ensure contextual relevance and responsiveness to emerging concerns. Internationally, while the findings may not be directly applicable, they offer valuable insights into the evolving landscape of childhood vaccine barriers in the post-pandemic context. The cross-sectional design conducted in the post-COVID-19 era may reflect pandemic-related challenges, such as heightened concerns about vaccine safety, mistrust, or disruptions in healthcare access, which could influence participants’ responses. Furthermore, the use of county to classify metro and non-metro areas may have resulted in misclassification, particularly in areas that blend rural, suburban, and urban characteristics.

Future research should address these limitations by adopting a longitudinal study design to reduce recall bias and track vaccination behaviors over time, possibly using clinical data. Randomized recruitment strategies involving a wider variety of settings and populations are required to mitigate selection bias and ensure a more representative sample. Expanding similar studies to other regions would also enhance generalizability, providing a clearer picture of how vaccination barriers vary across geographic and cultural contexts. Considering the post-COVID-19 context, future studies should explore the long-term effects of the pandemic on routine vaccine hesitancy, healthcare access, and public trust in vaccination. Finally, targeted interventions, informed by these findings, should be developed to address vaccine barriers in Tennessee.

## 5. Conclusions

The findings of this study are essential for shaping public health policies aimed at reducing vaccine hesitancy and improving equity in Tennessee. Addressing vaccine hesitancy requires targeted interventions that address concern, access, and importance barriers while acknowledging sociodemographic disparities. Special attention should be given to populations with the highest barriers, including lower-income families, uninsured parents, racial/ethnic minority groups, and parents with limited formal education. A sustained equity-driven approach that incorporates scientific communication, community engagement, and policy-level interventions is necessary to improve vaccine uptake and ensure equitable access to immunization in Tennessee.

## Figures and Tables

**Table 1:** Parental barriers to childhood vaccination in Tennessee: findings from the Searching for Hardships and Obstacles to Shots (SHOTS) survey, 2022–2024.

	Parental Barriers to Immunization	Mean	Standard Deviation	Cronbach’s Alpha
A	Immunization Access			0.941
1	I couldn’t get time off from work	0.39	0.97	
2	I didn’t know when my child needed to get his/her shots	0.33	0.87	
3	The clinic wait was too long	0.32	0.86	
4	I just forgot	0.29	0.83	
5	I didn’t have someone to take care of my other children	0.29	0.85	
6	The clinic/facility wasn’t open at a time I could go	0.28	0.82	
7	My child was sick and could not get his/her shots	0.28	0.77	
8	There were no appointments available at the clinic for shots	0.27	0.82	
9	The shots cost too much	0.23	0.79	
10	Getting my child in for shots is too much trouble	0.22	0.74	
11	I didn’t know where to take my child to get his/her shots	0.20	0.74	
12	I didn’t have a ride to the clinic	0.29	0.73	
B	Immunization Concern			0.943
1	If something bad happened to my child after a shot, I would feel like it is my fault	1.3	1.57	
2	I worry about what’s in the shot	0.96	1.36	
3	I worry my child might get sick from the shot	0.91	1.29	
4	I worry about how safe shots are	0.82	1.28	
5	I worry about the number of shots my child gets at one time	0.83	1.29	
6	I’m scared of the side effects of the shots	0.72	1.22	
C	Immunization Importance			0.920
1	I don’t think the shots work to prevent diseases	0.36	0.98	
2	I don’t think keeping my child up to date on shots is important	0.37	1.03	
3	I don’t think kids’ shots are important	0.36	1.0	
4	I don’t believe in getting kids shots	0.36	0.98	
5	My health care provider told me NOT to get my child his/her shots	0.21	0.77	

*Note. N* = 8954. The range of itemized questionnaires is 0–4, with 0 representing not at all a problem and 4 a very big problem.

**Table 2:** Distribution of sociodemographic characteristics of participants and parent reported under vaccinated children in TN, 2022–2024.

Sociodemographic Variables	Total Frequency (%) ^a^	Parent Reported Under Vaccinated Children (%) ^a^
Total	8954 (100)	317 (3.5)
Age of youngest child		
Early age (0–2 years)	1667 (19.1)	91 (5.5)
Preschool (3–6 years)	3406 (38.9)	113 (3.3)
School age (7–18 years)	3677 (42)	95 (2.6)
Education level of parents		
Less than high school	649 (7.3)	32 (4.9)
High school graduate	3543 (39.9)	115 (3.2)
Some college	2610 (29.4)	82 (3.1)
College graduate	2070 (23.3)	79 (3.8)
Race		
Non-Hispanic white	7327 (82.4)	230 (3.1)
Non-Hispanic Black	707 (8)	38 (5.4)
Hispanic	714 (8)	30 (4.2)
Asian/NHPI	62 (0.7)	6 (9.7)
^b^ American Indian/Indigenous	22 (0.2)	1 (4.5)
Middle Eastern or North African	16 (0.2)	0 (0)
Other/Multiracial	44 (0.5)	6 (13.6)
Household income level		
^c^ Up to 20K (0–99% FPL)	1664 (21.5)	74 (4.4)
21–40K (100–199% FPL)	1753 (22.6)	35 (2)
41–75K (200–400% FPL)	2308 (29.8)	96 (4.2)
76K and more (>400% FPL)	2032 (26.2)	64 (3.1)
Health insurance		
Has insurance	8312 (93.8)	254 (3.1)
No insurance	549 (6.2)	55 (10)
Rurality		
Non-metro	4641 (51.9)	159 (3.4)
Metro	4307 (48.1)	159 (3.4)

*Note*. ^a^ Column percent in the bracket; values in the first column represent the percentage of respondents in each sociodemographic group. Values in the second column represent the percentage of under-vaccinated children within each sociodemographic group. ^b^ American Indian, Native American, Alaska Native, or Indigenous; ^c^ FPL = income data were recategorized based on the Federal Poverty Level (FPL) for a two-member household, regardless of actual household size, with adjustments made to align original categories with FPL classifications.

**Table 3:** Association of sociodemographic variables with vaccine barriers (access, concern, importance) in Tennessee, 2022–2024.

Sociodemographic Variables	Access Barrier	Concern Barrier	Importance Barrier	Total SHOT Score
	Median (IQR, Mean)	Median (IQR, Mean)	Median (IQR, Mean)	Median (IQR, Mean)
Age of youngest child	<0.001 *	0.053	0.368	0.003 *
Early age (0–2 years)	0 (0–3, 3.4) ^a^	2 (0–10, 5.9)	0 (0–0, 1.8)	4 (0–17, 11.1) ^a^
Preschool (3–6 years)	0 (0–3, 3.3) ^a^	2 (0–10, 5.4)	0 (0–0, 1.6)	4 (0–17, 10.3) ^a^
School age (7–18 years)	0 (0–3, 3.2) ^b^	2 (0–10, 5.4)	0 (0–0, 1.6)	4 (0–17, 10.2) ^b^
Education level of parents	<0.001 *	0.06	<0.001 *	0.023 *
Less than high school	0 (0–4, 4.1) ^a^	1 (0–8, 5.0)	0 (0–1, 1.9) ^a^	3 (0–16, 11.0) ^ab^
High school graduate	0 (0–3, 3.8) ^a^	2 (0–10, 5.7)	0 (0–1, 1.9) ^a^	4 (0–16, 11.4) ^a^
Some college	0 (0–3, 3.1) ^b^	2 (0–9, 5.4)	0 (0–0, 1.6) ^a^	4 (0–15, 10.2) ^ab^
College graduate	0 (0–2, 2.3) ^b^	2 (0–9, 5.3)	0 (0–0, 1.3) ^b^	4 (0–13, 8.9) ^b^
Race	<0.001 *	0.006 *	<0.001 *	<0.001 *
Non-Hispanic white	0 (0–2, 3.0) ^a^	2 (0–9, 5.4) ^a^	0 (0–12, 1.5) ^a^	4 (0–14, 10.0) ^a^
Non-Hispanic Black	0 (0–4, 4.5) ^b^	3 (0–11.8, 6.2) ^ab^	0 (0–2, 2.1) ^b^	4 (0–20, 12.8) ^b^
Hispanic	0 (0–5,4.7) ^c^	2 (0–9, 5.2) ^a^	0 (0–1, 2.1) ^b^	5.5 (0–17.8, 11.9) ^b^
Asian/NHPI	0 (0–10, 6.1) ^c^	4 (0–13, 7.3) ^ab^	0 (0–5.5, 3.4) ^c^	10 (0–27, 16.9) ^bc^
^d^ American Indian/Indigenous	1 (0–5, 4.5) ^c^	4 (0.5–18.5, 8.2) ^ab^	0 (0–4.5, 2.8) ^bc^	5 (1.5–23.5, 15.5) ^bc^
Middle Eastern or North African	1.5 (0–11, 10.5) ^c^	3 (0–15.5, 7.3) ^ab^	0 (0–10.25, 4.9) ^c^	11.5 (0–22.5, 22.7) ^bc^
Other/Multiracial	4 (0–12, 7.7) ^c^	8 (0–20, 10) ^b^	0 (0–12, 5.8) ^c^	21 (1–40, 23.7) ^c^
Household income level	<0.001 *	0.062	<0.001 *	<0.001 *
^e^ Up to 20K (0–99% FPL)	0 (0–4, 4.5) ^a^	2 (0–10, 5.6)	0 (0–11, 2.2) ^a^	4 (0–16, 12.3) ^a^
21–40K (100–199% FPL)	0 (0–5, 3.8) ^a^	2 (0–10, 5.6)	0 (2–15, 1.7) ^b^	5 (0–18, 11.1) ^a^
41–75K (200–400% FPL)	0 (0–2, 3.1) ^b^	2 (0–10, 5.7)	0 (0–12, 1.6) ^b^	4 (0–15, 10.4) ^a^
76K and more (>400% FPL)	0 (0–1, 2.1) ^c^	2 (0–9, 5.2)	0 (0–10, 1.4) ^c^	3 (0–12, 8.7) ^b^
Health insurance	<0.001 *	0.441	<0.001 *	0.007 *
Has insurance	0 (0–3, 3.2)	2 (0–9, 5.5)	0 (0–0, 1.6)	4 (0–15, 10.4)
No insurance	0 (0–4, 4.2)	2 (0–10, 5.9)	0 (0–3, 2.5)	6 (0–20, 12.6)
Rurality	<0.001 *	0.925	0.046 *	0.089
Non-metro	0 (0–2, 3.1)	2 (0–9, 5.5)	0 (0–12, 1.6)	4 (0–14, 10.2)
Metro	0 (0–3, 3.6)	2 (0–9, 5.6)	0 (0–12, 1.8)	4 (0–16, 10.9)

*Note*. Reported values are medians (IQR, mean) for each age group. The means are provided solely as a supplementary estimate to illustrate relative differences in barrier levels across groups. *p*-values are derived from the Kruskal–Wallis test, which was used to compare medians across multiple independent groups. (*) indicate statistically significant differences at *p* < 0.05. Groups sharing the same superscript (e.g., ‘a’ to ‘e’) are not significantly different from each other. Groups labeled with multiple superscripts (e.g., ‘ab’) are statistically similar to both corresponding groups. ^d^ American Indian, Native American, Alaska Native, or Indigenous. ^e^ FPL = income data were recategorized based on the Federal Poverty Level (FPL) for a two-member household, regardless of actual household size.

**Table 4:** Relationship between up-to-date vaccination status of children and vaccine barriers (access, concern, importance) in Tennessee, 2022–2024.

Parental Vaccine Barriers	B (Coefficient)	aPR	95% CI	*p*-Value
Access Barrier	0.001	1.001	[1.00 1.002]	<0.001
Concern Barrier	−0.002	0.998	[0.997 0.998]	<0.001 *
Importance Barrier	−0.003	0.997	[0.996 0.998]	<0.001 *

*Note*. For the binary regression for the outcome of up-to-date vaccination, the model was adjusted for race, family income, health insurance, and education level of parents. (N = 8963), aPR = adjusted prevalence ratio. (*) indicate statistically significant *p* < 0.05.

## Data Availability

Data will be made available on request.
